# The Impact of Ischemia/Reperfusion Injury on Liver Allografts from Deceased after Cardiac Death versus Deceased after Brain Death Donors

**DOI:** 10.1371/journal.pone.0148815

**Published:** 2016-02-10

**Authors:** Jin Xu, Blayne Amir Sayed, Ana Maria Casas-Ferreira, Parthi Srinivasan, Nigel Heaton, Mohammed Rela, Yun Ma, Susan Fuggle, Cristina Legido-Quigley, Wayel Jassem

**Affiliations:** 1 Liver Transplant Unit, Institute of Liver Studies, King’s College Hospital, London, United Kingdom; 2 Institute of Pharmaceutical Science, Faculty of Life Science & Medicine, King’s College London, London, United Kingdom; 3 Department of Transplant Surgery, Emory University, Atlanta, Georgia, United States of America; 4 Department of Analytical Chemistry, Nutrition and Food Science, University of Salamanca, Salamanca, Spain; 5 Nuffield Department of Surgery, University of Oxford, John Radcliffe Hospital, Oxford, United Kingdom; Medical University of Graz, AUSTRIA

## Abstract

**Background and aims:**

The shortage of organs for transplantation has led to increased use of organs procured from donors after cardiac death (DCD). The effects of cardiac death on the liver remain poorly understood, however. Using livers obtained from DCD versus donors after brain death (DBD), we aimed to understand how ischemia/reperfusion (I/R) injury alters expression of pro-inflammatory markers ceramides and influences graft leukocyte infiltration.

**Methods:**

Hepatocyte inflammation, as assessed by ceramide expression, was evaluated in DCD (n = 13) and DBD (n = 10) livers. Allograft expression of inflammatory and cell death markers, and allograft leukocyte infiltration were evaluated from a contemporaneous independent cohort of DCD (n = 22) and DBD (n = 13) livers.

**Results:**

When examining the differences between transplant stages in each group, C18, C20, C24 ceramides showed significant difference in DBD (p<0.05) and C22 ceramide (p<0.05) were more pronounced for DCD. C18 ceramide is correlated to bilirubin, INR, and creatinine after transplant in DCD. Prior to transplantation, DCD livers have reduced leukocyte infiltration compared to DBD allografts. Following reperfusion, the neutrophil infiltration and platelet deposition was less prevalent in DCD grafts while cell death and recipients levels of serum aspartate aminotransferase (AST) of DCD allografts had significantly increased.

**Conclusion:**

These data suggest that I/R injury generate necrosis in the absence of a strong inflammatory response in DCD livers with an appreciable effect on early graft function. The long-term consequences of increased inflammation in DBD and increased cell death in DCD allografts are unknown and warrant further investigation.

## Introduction

The increasing demands for suitable organ donors for liver transplantation exceed the number of donors which has remained largely static [1]. The worsening organ shortage is reflected in the median time to transplant in wait-listed adult patients. In the United States median time increased from 14.8 months in 2004 to 19.5 months in 2011. As such, transplant centres and allocation organizations have attempted to expand the pool of acceptable donors, including the use of DCD donors [2]. Although some centres have reported good results by using DCD allografts, other data indicates that recipients of controlled DCD liver allografts have an increased incidence of graft dysfunction, early graft loss and cholangiopathy as compared to recipients of DBD livers [3–7].

The pathophysiology of cardiac death is markedly different from that of brain death. As compared to livers obtained from DBD, in which there is no consistent proceeding cardiac arrest, DCD livers are subjected to additional hypoxic insult. However, brain death generates an inflammatory response with the release of various pro-inflammatory mediators, leading to upregulated expression of adhesion molecules on vascular endothelium and subsequent leukocyte tissue infiltration [8–10]. In a previous study, we demonstrated that prior to transplantation DCD allografts have lower expression of ICAM-1, potentially suggesting less allograft inflammation [11].

Ischemia/reperfusion (I/R) injury is associated with the release of reactive oxygen species and pro-inflammatory mediators [12], and there is evidence to suggest that brain death followed by I/R injury synergistically aggravates the insult associated with cold storage [10, 13]. Elevated levels of ceramides, sphingolipid molecules associated with I/R injury, promote inflammation and downstream apoptosis by enhancing susceptibility to palmitate-induced cell death [14–16]. The role of ceramides has yet to be described in human liver transplantation [15] despite previous observations of ischemia/reperfusion-induced accumulation of ceramides in various organs, including the liver [14, 16].

**Fig 1:**
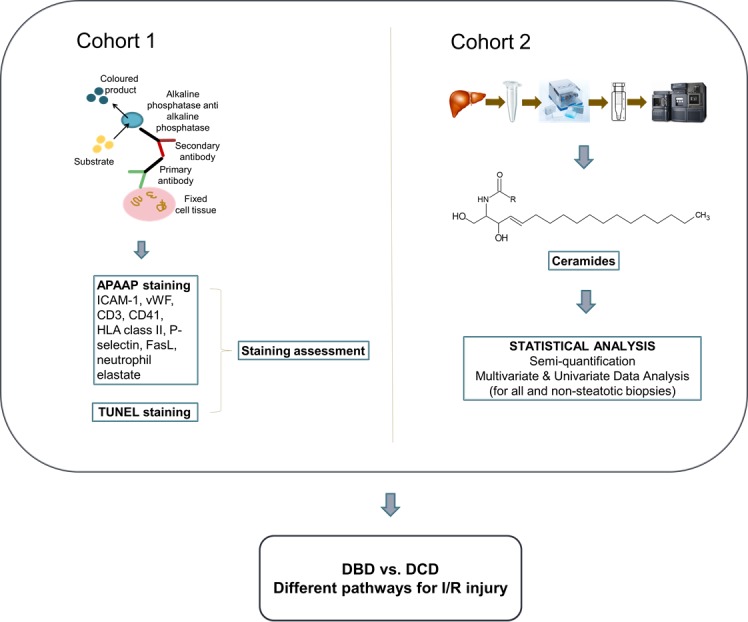
Study design. Two independent cohorts of immunohistochemistry and targeted lipids analysis found that DBD and DCD liver allografts have different pathways for I/R injury.

In this study we assessed the impact of I/R on DCD as compared to DBD liver allografts by assessing leukocyte infiltration, expression of pro-inflammatory molecules, including ceramides, and cell death before and after reperfusion (study design as seen in **Fig 1**).

## Patients and Methods

### Patient sampling

Demographic and clinical data of donors and recipients were drawn from donor offer data and hospital records. Cohort 1 with DCD (n = 13) and DBD (n = 10) biopsies were applied to assess ceramide expression. Cohort 2 with DCD (n = 22) and DBD (n = 13) allografts were evaluated for immunohistochemistry assessment.

Tru-cut biopsies were taken prior to and approximately 1 hour ± 15min following reperfusion. All biopsies were immediately snap-frozen in liquid nitrogen and transferred to –80°C until the time of processing. Details about the sample collection were provided in **supporting information (S1 File)**. Donor clinical data (**Table 1**) and recipient clinical data (**Table 2**) include both cohorts. The study was approved by the Ethics Committee of King’s College Hospital. All retrievals and transplant were performed by King’s College Hospital transplant team, and only adults transplant (age>18) were considered for this study. Consent was obtained from all patients/their next of kin for collection of data and they were given the option to withdraw written consent freely, and all clinical investigation has been conducted according to the principles expressed in the Declaration of Helsinki. None of the transplant donors were form a vulnerable population and all donors or next of kin provided written informed consent that was freely given. Patient information was anonymized prior to use in this study.

**Table 1:** Summary of clinical data for liver donors.

**Cohort 1**		**DBD**	**DCD**	***p*-value**^**[b]**^
		(N = 10)	(N = 13)	**DBD vs DCD**
Age(years)		51(19)	54(13)	0.69
Gender(female/male)		7/3	7/6	0.67
	No	5	9	
Hepatic steatosis	Mild (<30%)	2	4	0.13
	Moderate (30–60%)	3	0	
GGT (IU/L) ^[a]^		35(14)	28(19)	0.73
AST (IU/L) ^[a]^		39(6)	70(53)	1
Bilirubin (μmol/L) ^[a]^		14(13)	8(4)	0.79
ITU stay (days)		5(9)	2(2)	0.48
Inotrop support (Y/N)		7/3	9/4	1
WIT (min)		NA	20(7)	
CIT (min)		496(212)	389(117)	0.60
**Cohort 2**		DBD	DCD	*p*-value^[b]^
		(N = 13)	(N = 22)	**DBD vs DCD**
Age(years)		33(17)	39(15)	ns
Gender(female/male)		7(6)	10(12)	ns
ITU stay (hours)		53(47)	72(51)	ns
Inotrop support (Y/N)		11/2	15/7	0.43
	No	8	12	
Hepatic steatosis	Mild (<30%)	5	8	0.74
	Moderate (30–60%)	0	2	
WIT (min)		NA	14(5)	
CIT (min)		671(177)	477(112)	0.02
AST (IU/L) ^[a]^		87(10)	65(9)	ns

DBD, donation after brain death; DCD, donation after circulatory death; GGT, gamma-glutamyl transferase; AST, aspartate aminotransferase; ITU, intensive therapy unit; WIT, warm ischemia time; CIT, cold ischemia time.

Continuous values are expressed as means (standard deviation).

[a] Tested on the day of operation

[b] Mann Whitney test or Fisher exact test.

ns: not significant; NA, not applicable.

**Table 2:** Summary of clinical data for liver recipients.

**Cohort 1**	**DBD**	**DCD**	***p*-value**^**[b]**^
	(N = 10)	(N = 13)	**DBD vs DCD**
Age (years)	42(13)	57(6)	0.56
Gender (female/male)	7/3	4/9	0.10
BMI (kg/m^2^)	24(4)	26(5)	0.34
MELD Score (median)	12	13	1.0
Alcoholic liver disease (ALD)	2	9	
Primary sclerosing cholangitis (PSC)	2	0	
Hepatic C virus (HCV)	1	5	
Hepatocellular carcinoma (HCC)	0	2	0.07
Biliary atresia (BA)	0	0	
Others	5	1	
AST (IU/L) ^[a]^	963(611)	2356(2714)	0.17
Bilirubin day 5 (μmol/L)	80(74)	74(70)	0.92
INR day 2	1.43(0.16)	1.51(0.46)	0.65
One year audit report (survival rate)	100%	81.8%	NA
**Cohort 2**	DBD	DCD	*p*-value^[b]^
	(N = 13)	(N = 13)	**DBD vs DCD**
Age (years)	44(17)	43(15)	ns
Gender (female/male)	9/4	7/6	ns
Alcoholic liver disease (ALD)	2	1	
Primary biliary cirrhosis (PBC)	2	2	
Hepatic C virus (HCV)	2	4	
Cryptogenic	4	1	0.73
Autoimmune hepatitis	2	3	
Hepatic B virus (HBV)	1	2	

DBD, donation after brain death; DCD, donation after circulatory death; BMI, body mass index; MELD, model for end-stage liver disease; ALD, alcoholic liver disease; PSC, primary sclerosing cholangitis; HCV, hepatitis C virus; HCC, hepatocellular carcinoma; BA, biliary atresia; AST, aspartate aminotransferase; INR, international normalized ratio.

Continuous values are expressed as means (standard deviation).

^[a]^ Maximum value during 14-day period

^[b]^ Mann Whitney test or Fisher exact test.ns: not significant; NA, not applicable.

### Ceramide analysis

Liquid chromatography-mass spectrometry (LC-MS) analysis was performed using a previously published method [17]. The identification of ceramides Cer16, Cer18, Cer20, Cer22 and Cer24 (with the number suffix denoting the length of the acyl chain) was achieved by structure and fragmentation patterns comparison of the MS^2^ data with a C8 ceramide standard and literature [18–21]. Five ceramides were measured in the LC-MS data using Waters Mass Lynx software (Waters Corporation, Milford, MA) and their peak area ratios to internal standard were calculated. Mean ratio values were used to plot the heat-map using an open source ‘R’ package, 'gplots' [22]. Subsequently univariate non-parametric Mann Whitney test was performed to examine the mean difference of each ceramide level in each group at pre- and post-transplantation stage, as well as between DBD and DCD at both stages.

### APAAP staining

Approximately 7μm cryostat sections were air-dried and fixed with acetone for 10 minutes at room temperature and stained using an alkaline phosphatase anti-alkaline phosphatase (APAAP) staining technique. Sections were incubated with monoclonal antibodies neutrophil elastase (NP57, neutrophils), ICAM-1 (6.5B5), Von Willebrand Factor (F8/86) and VCAM-1 (1.4C3) CD3 (UCHT-1, T lymphocytes) (DAKO Ltd, High Wycombe, UK), HLA class II (NFK1, monomorphic HLA-DR, DP)[23] and P-selectin (AK4), CD41 (MWReg30, anti-platelets) and FasL (G247-4) (Pharmingen, Ltd, San Diego, USA). More details about staining procedures are provided in **S1 File**.

### TUNEL staining

TUNEL technique was performed to detect apoptosis *in situ* using R&D systems kit. Frozen liver tissue sections were deparaffinised and rehydrated through three changes between xylene and graded alcohol, then washed in PBS for 5 min, and incubated in 20 lg/mL proteinase K for 15 min at room temperature. The DNA fragmentation detection Kit Colorimetric-TdT Enzyme was used according to the manufacturer’s instructions. TUNEL staining was assessed by cell counting of positive cells. 2 sections from each biopsy were stained and counted and between10-20 fields were assessed. Only positively stained hepatocytes were considered and the mean of cell counting was calculated.

### Assessment of staining

The percentage area of CD3, CD41, neutrophil elastase, P-selectin and FasL expressions were quantitated by morphometric point counting as previously described [11]. Details of assessment methods were included in the **S1 File**.

### Statistical analysis

The results of the immunohistochemistry findings were analysed with respect to donor, Intensive Care Unit (ICU) parameters using Chi-square, Student’s *t-*test and Mann Whitney U for proportions and means, and Spearman’s test to determine significant correlations.

For ceramide analysis, spearman’s correlation analysis was applied separately in DCD and DBD groups to investigate the correlation of ceramides at both pre & post-transplant with recipients’ 14-day clinical data. All obtained *p* values were adjusted for multiple comparisons using Benjamini and Hochberg correction to control the false discovery rate (FDR) [24]. OPLS-DA (SIMCA 13.0.2, Umetrics, Sweden) was used for multivariate analysis using the 5 ceramides as variables. Two models were investigated, DBD vs. DCD (n = 46), and DBD vs. DCD for liver biopsies from non-steatosis (n = 28). Statistical calculations were conducted in SPSS 22 (IBM: Armonk, United States).

## Results

### Clinical outcomes

There were no significant differences in donor and recipients clinical parameters between the two groups (**Tables 1 & 2**) or post-transplant biochemical outcome.

### Multivariate analysis (OPLS-DA) for ceramides at per transplant stage

**Fig 2:**
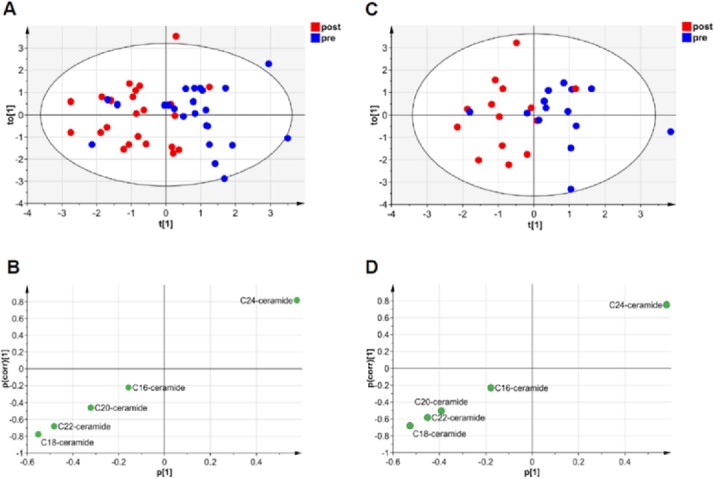
Multivariate analysis of pre vs. post biopsies and ceramides distribution. (A) OPLS-DA score plot visualizing the grouping of pre vs. post for all biopsies. (B) S-plot illustrating ceramides correlation to groups for model in A. (C) OPLS-DA score plot showing group separation between pre- and post-transplant for none steatotic grafts. (D) S-plot indicating ceramides correlation to groups for model in C.

OPLS-DA model (**Fig 2**) between pre and post-transplant grafts was built according to the selected 5 ceramides (**Table A in S1 File**), Cer (34:1), Cer (36:1), Cer (38:1), Cer (40:1) and Cer (42:1).

The score plot (**Fig 2A**) showed separation of these two transplant stages, the model’s figures of merit were R^2^X = 0.89. R^2^X explains a data percentage (89%) which is explained by this model. The S-plot in **Fig 2B** indicated that Cer24 correlated with pre transplant stage and the other four ceramides correlated with post transplantation. Cer18 and Cer24 showed the highest correlation (p(corr) = 0.8) with post and pre transplantation stage, respectively.

Similarly, the OPLS-DA model for non-fatty biopsies in **Fig 2C** addressed figures of merit with R^2^X = 0.88, while Cer18 correlated most with post-transplant in the S-plot (p(corr) = 0.7) and Cer24 correlated with pre-transplant (p(corr) = 0.8) (**Fig 2D**).

### Univariate analysis for ceramides per donor type during transplant

**Fig 3:**
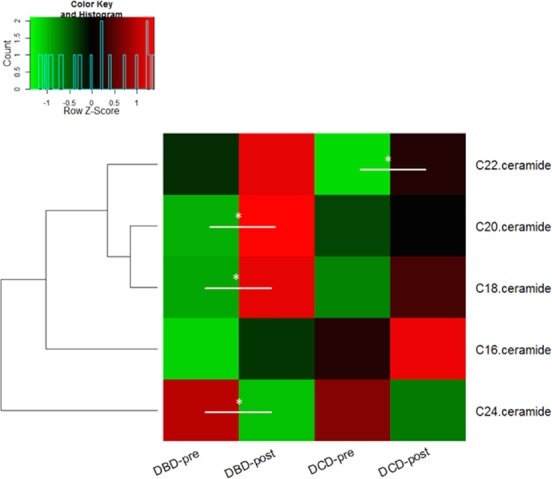
Heat-map showing distinct ceramides profiles of DBD and DCD tissue in 46 transplant samples. Values are mean amounts per donor group at pre and post-transplantation stages. A clustering analysis (dendrogram) shows which lipids differ most; red depicts increased amounts and green decreased.

The amounts for the 5 selected ceramides were measured and plotted in a heat-map dendrogram (**Fig 3**). When comparing DBD-pre to DCD-pre, no significant difference was observed for all five ceramides, and the same result applies to DBD-post and DCD-post.

To assess the impact of ischemia injury, all 46 biopsies and their ceramides levels were compared between pre- and post-transplantation within each donor group. Four ceramides were found altered in DBD at post-transplantation (higher Cer18, Cer20, Cer22 and lower Cer24) (p<0.05) while one ceramide, Cer24, decreased in DCD post-transplantation (p<0.05).

### Correlation of ceramides to clinical data

Spearman’s rank correlation analysis was applied separately in DCD and DBD groups to investigate the correlation of ceramides at both pre & post-transplant with recipients’ 14-day clinical data. According to the correlation result, creatinine, INR, bilirubin, donor age and WIT show high significance, then Benjamini and Hochberg false discovery correction was used to test the p value of those 5 parameters. After the p-value correction, Cer18 showed significant correlation to bilirubin and INR at pre-transplantation, creatinine at post-transplantation in DCD, while no significant correlation reflect in DBD group at both pre and post-transplantation (see details in **Table B in S1 File**).

### Graft leukocyte infiltration, activation marker expression and cell death prior to transplantation

**Fig 4:**
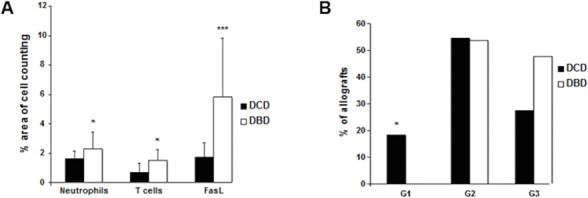
Immunohistochemistry results prior to transplantation. (A) Leukocyte infiltration and FasL expression in liver allografts prior to transplantation. Biopsies from DBD and DCD liver biopsies were stained with monoclonal antibodies against neutrophil elastase, CD3 and FasL. (B) Expression of ICAM-1 in liver allografts prior to transplantation.

DCD allografts had significantly lower levels of neutrophil and T cell infiltration compared to DBD livers (1.6 ± 0.5 vs. 2.3 ± 1.1; *p* = 0.03 and 0.7 ± 0.6 vs.1.5 ± 0.7; *p* = 0.02 respectively) (**Fig 4A**). The tumour necrosis factor (TNF)-family member activation marker Fas ligand (FasL) was expressed at a much lower level in DCD as compared to DBD allografts (1.7 ± 1 vs. 5.8 ± 4; *p*<0.001) (**Fig 4A**).

**Fig 5:**
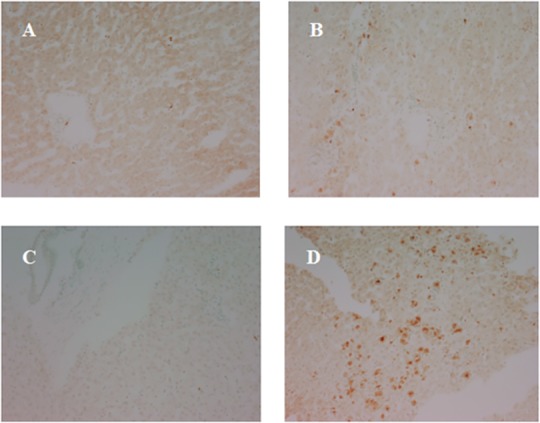
Representative TUNEL staining in pre- and post-reperfusion biopsies in DBD and DCD livers. (A) pre-reperfusion biopsies in DBD, (B) post-reperfusion biopsies in DBD, (C) pre-reperfusion biopsies in DCD, (D) post-reperfusion biopsies in DCD.

There were similar number of allografts expressing intermediate levels of ICAM-1 (54% of DCD and 53% of DBD expressing grade 2) (**Fig 4B**). However, only 27% of DCD livers (6/22) as compared to 47% of DBD livers (6/13) expressed high levels of ICAM-1 (grade 3). While none of the DBD allografts expressed low levels of ICAM-1 (grade 1), 18% of DCD allografts (4/22) demonstrated low levels of ICAM-1 (grade 1). P-selectin, which is expressed on activated platelets, was expressed at significantly lower levels in DCD as compared to DBD allografts prior to transplantation (0.24 ± 0.3 vs. 0.57 ± 0.5; *p* = 0.04) (0.81 ± 0.7 vs. 2.7 ± 1; *p*< 0.001). The platelet marker CD41 also was expressed at lower levels in DCD compared to DBD allografts (0.81 ± 0.7 vs. 2.7 ± 1; *p*< 0.001), and platelets appeared to be primarily deposited on the sinusoidal endothelium. Finally, expression of Von Willebrand factor (vWF), a pro-coagulant factor released by activated endothelium, was lower in DCD compared to DBD livers. Of 13 DCD livers 11 (85%) expressed grade 0 and 2 allografts (15%) grade 1 of vWF staining. In contrast 5 out 12 DBD livers (42%) expressed vWF at grade 0 (*p* = 0.02), 6 (50%) at grade 1 and 1 (8%) as grade 2 (*p* = 0.04). Terminal deoxynucleotidyl transferase dUTP nick end labelling (TUNEL) staining, which detects apoptotic cells, demonstrated low levels of cell death in the pre-transplantation biopsies in both DCD and DBD livers with no significant differences between the two groups (1.1 ± 1.4 vs. 1.8 ± 2.8 cell per field; *p* = 0.7) (**Fig 5A and 5C**).

### Graft function, leukocyte infiltration, activation marker expression and cell death following reperfusion

**Fig 6:**
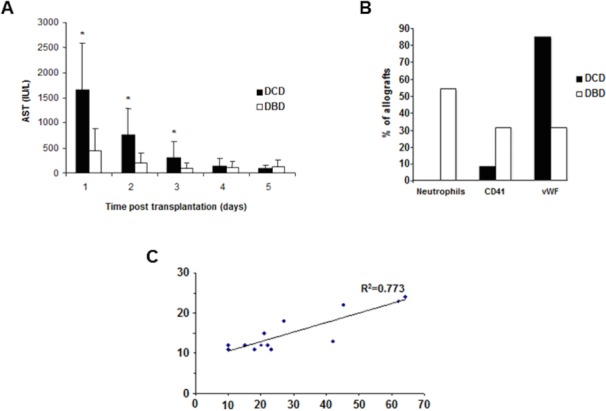
Immunohistochemistry results following reperfusion. (A) Recipient serum levels of aspartate aminotransferase (AST) following transplantation. (B) Neutrophil infiltration, platelet activation and expression of vWF post-reperfusion. (C) Correlation between post-reperfusion cell death and WIT.

Recipients of DCD allografts had significantly higher AST levels in the first three days following transplantation compared to the DBD group (**Fig 6A)**.

Comparison of the expression of neutrophil elastase, CD41, P-selectin and vWF from DCD and DBD pre- and post-reperfusion liver biopsies were made to determine if any measurable differences existed. None of the DCD livers demonstrated increased in neutrophil infiltration in post- versus pre-reperfusion biopsies (*p* = 0.01). In contrast, approximately 54% of DBD allografts had significantly increased neutrophil infiltration post- reperfusion as compared to pre-reperfusion DBD allografts (**Fig 6B**). Neutrophil infiltration was observed predominantly in sinusoids and portal tracts. Only 8% (1/13) of DCD livers had increased platelet deposition post-reperfusion while 31% (4/13) of DBD livers had increased platelet deposition, but this difference was not statistically significant (p = 0.3). The pattern of vWF was largely reversed, with 85% (11/13) of DCD livers demonstrating an increase in expression compared to 31% (4/13) of DBD grafts (p = 0.01). The expression of vWF was predominately located on sinusoidal endothelium. There were no significant differences in expression of adhesion molecules. However, direct comparison of DCD and DBD allografts following reperfusion revealed significantly higher levels of cell death in the DCD allografts versus their DBD counterparts (30 ± 19 vs. 4.4 ± 4 cells per field; p = 0.001). Histological analysis demonstrated that cell death occurred predominantly in hepatocytes and sinusoidal endothelium (**Fig 5**) and that there was a correlation between duration of WIT and cell death in DCD livers (**Fig 6C**).

### Correlation of pro-inflammatory molecules with donor clinical parameters

In DCD livers high levels of neutrophil infiltration and CD41 deposition were correlated with traumatic head injury in the donor (R: 0.570, *p* = 0.04 and R: 0.632, *p* = 0.02 respectively). High levels of CD41 deposition were correlated with extended CIT (R: 0.729, *p* = 0.005). Increased levels of neutrophil infiltration and expression of ICAM-1, vWF and CD41 were associated with extended ICU stay of the DCD donors (R: 0.783, *p* = 0.002; R: 0.818, *p* = 0.001; R: 0.703, *p* = 0.01; R: 0.708, *p* = 0.007 respectively). Finally, prolonged WIT correlated with increased levels of cell death following transplantation (R: 0.712, *p* = 0.01). There was no other significant association between post-reperfusion inflammatory events and transplant outcome.

## Discussion

The increasing demand for liver allografts combined with largely static organ donation rates lead to the broadening of donor inclusion criteria. One area of intense interest is the use of DCD allografts, which have a prolonged period of WIT [25]. Data indicate that patients receiving DCD allograft are at higher risk of biliary complications, graft failure and patient mortality [4], and although outcomes can be improved with careful donor selection, they are not as favourable as with DBD allografts [3, 6, 7]. To better understand the molecular mechanisms of worsened DCD graft function we compared the expression of various inflammatory mediators at pre-and post-perfusion in DCD and DBD allografts.

Ceramides are well known markers of inflammation and elevated ceramide level are thought to produce inflammation and downstream apoptosis by enhancing susceptibility to palmitate-induced cell death [15]. During I/R injury, accumulation of ceramides was observed in tissues, however it is not yet known how the size of a ceramide determines inflammation response [14, 16]. In-vitro experiments showed that high level of C16-ceramide resulted in TNF-α-induced hepatocyte apoptosis [26]. Conversely elevated de-novo C18-ceramide, synthesised by CerS4 in the liver, was also found to function as a powerful pro-apoptotic activator in tumour cells [27, 28]. Bigger ceramides also called very long chain ceramides (C24-ceramides) are thought to be the most abundant in healthy liver and their decrease can in turn indicate liver pathology [29].

In this study five ceramides of increasing acyl chain from long to very long chain: C16, C18, C20, C22 and C24 were measured. These results are in line with what was expected from IR response, increased long chain (C18-C20) and decreased very long chain (C24) suggesting higher inflammation at post-transplant stage. C22 increased its level after graft implantation; this is not in agreement with an inflammation response considering it is also a very long chain ceramide. Differences between DBD and DCD were insignificant, however overall changes in DBD from pre- to post transplantation for C18, C20, C24 (p<0.05) were significant and C22 (p<0.05) were more pronounced for DCD suggesting that the inflammation response was more severe in DBD.

Lang *et al*. found that bilirubin induces ceramide formation [30], which explains the high correlation of bilirubin and Cer18 at DCD pre-transplant stage in our study. Moreover, as bilirubin is an antioxidant that can stimulate apoptosis of various cells [31, 32], the higher Cer18 level in DCD at pre-transplant stage compared with DBD indicates that severe apoptosis took place in DCD hepatocytes. INR, the assessment parameter for coagulation monitoring [33], indicated the liver injury when its value is elevated, hence, the correlation of INR with Cer18 in DCD post-transplant demonstrates ischemia/reperfusion injury in DCD. The association between C18-ceramide and serum level of creatinine is of interest as renal dysfunction post-transplant has been seen to occur at higher rate in liver transplantation from DCD [34].

Significantly higher levels of neutrophil, T cell infiltration, and ICAM-1 expression were detected in DBD compared to DCD liver allografts prior to transplantation. This pattern fits with data from animal and human studies that demonstrates that brain death (BD) results in a systemic inflammatory response in the donor with the release of various cytokines and chemokines [9, 35]. Production of these pro-inflammatory molecules leads to increased expression of adhesion molecules on the endothelium and subsequent leukocyte infiltration and damage to parenchymal cells.

Increased neutrophil migration and platelet activation in DBD livers correlates with donor cerebral ischemia as reported in both renal and liver transplantation, and the level of hepatic inflammation is also associated with traumatic brain injury [11, 36]. Our results demonstrated increased platelet adhesion and vWF expression prior to reperfusion in DBD livers, and the extent of platelet adhesion correlated with prolonged CIT. Platelet adhesion to hepatic sinusoidal endothelium induces release of P-selectin and vWF and prior to transplantation has been correlated to CIT duration and graft function in both animal models and human liver transplantation [37–39]. As extended cold preservation damages endothelial cells, these results may also reflect the shorter cold storage times for DCD allografts [40].

In an animal model, the inflammation associated with brain death plays a synergistic role with extended CIT leading to a more vigorous I/R injury [13]. Increased neutrophil infiltration in DBD livers likely reflects more effective transmigration due to elevated ICAM-1 expression pre-reperfusion. However, although DBD allografts had increased neutrophil infiltration and platelet deposition post-reperfusion, there was relatively consistent expression of adhesion molecules between DBD and DCD allografts. Interestingly, DCD livers upregulate vWF post-reperfusion more substantially compared to DBD livers. The etiology of this upregulation is unclear but is potentially related to the warm ischemic process unique to DCD allografts. Limited animal work demonstrated that warm ischemia associated with I/R injury in the gut induces upregulation of vWF expression [41]. The consequences of the increased endothelial vWF expression, for platelet binding or otherwise, were not immediately evident.

We also demonstrated a significantly higher level of cell death in DCD livers following reperfusion, which correlated to the duration of WIT. Hepatocyte apoptosis and liver necrosis are common features of I/R injury in the liver [42]. Duration of WIT significantly contributes to the extent of cell death in animal models and human liver transplantation [43, 44]. Warm ischemia primes the cellular intrinsic pathway of apoptosis by reducing hepatocyte antioxidant capabilities, priming mitochondria to produce reactive oxygen species upon reperfusion and contributing to the development of mitochondrial permeability. This subsequently leads to the release of pro-apoptotic and -necrotic factors, the former activated by the caspase cascade in programmed cell death [12, 45]. Conversely, upregulated FasL, an important regulator of the extrinsic apoptotic pathway, potentially accounts for the cell death observed in DBD livers, which has been previously described [46]. Ceramides, which activate the NLRP3 inflammasome, were also more elevated in DBD allografts, again potentially indicating Fas-FasL mediated apoptosis.

In conclusion, as compared to DBD allografts, DCD grafts appear to have reduced leukocyte infiltration before and after transplantation. Ceramides can be used as indicator of inflammation for liver transplantation. However, DCD livers have a higher rate of cell death that correlates with WIT and post-transplant serum AST levels, suggesting that DCD livers are prone to necrosis rather than inflammation.

## Supporting Information

S1 File Identification of markers based on molecular weight, retention time and collision induced dissociation fragmentation of lipids (**Table A**). Correlation of ceramides (n = 23) to clinical outcomes (**Table B**).
(DOCX)

## Abbreviations

APAAP: Alkaline phosphatase-anti-alkaline phosphatase
DBD: Deceased after brain death
DCD: Deceased after cardiac death
CIT: Cold ischemia time
HLA: Human leukocyte antigen
ICAM-1: Intracellular adhesion molecule -1
ICU: Intensive care unit
UW: University of Wisconsin
VCAM-1: Vascular cell adhesion molecule -1
vWF: Von Willebrand factor
WIT: Warm ischemia time
