# Supplementary material for: The Impact of Ischemia/Reperfusion Injury on Liver Allografts from Deceased after Cardiac Death versus Deceased after Brain Death Donors
Source: PLoS One. 2016 Feb 10;11(2):e0148815. doi: 10.1371/journal.pone.0148815 (PMC4749185; doi:10.1371/journal.pone.0148815)
Supplement: S1 File — Identification of markers based on molecular weight, retention time and collision induced dissociation fragmentation of lipids (Table A). Correlation of ceramides (n = 23) to clinical outcomes (Table B). (DOCX) [file pone.0148815.s001.docx]

**Supporting Information for:**

The impact of ischemia/reperfusion injury on liver allografts from deceased after cardiac death versus deceased after brain death donors

Jin Xu*, Blayne Amir Sayed, Ana Maria Casas-Ferreira, Parthi Srinivasan, Nigel Heaton, Mohammed Rela, Yun Ma, Susan Fuggle, Cristina Legido-Quigley, Wayel Jassem*

*Corresponding authors

E-mail: [jin.xu@kcl.ac.uk](mailto:jin.xu@kcl.ac.uk) (JX), E-mail: [wayel.jassem@kcl.ac.uk](mailto:wayel.jassem@kcl.ac.uk) (WJ)

**Sampling**

Twenty-two Maastricht category III controlled DCD allografts, 13 of which were used for transplantation, and 13 contemporaneous matched DBD liver transplants were used for all immunohistochemistry analysis. For ceramide analysis, 23 paired DCD and DBD allografts obtained during the same time period were utilized. All patients were transplanted at King’s College Hospital. The technique of controlled DCD retrieval includes a “standoff” period of 5-10 minutes after cardiac arrest prior to initiation of organ procurement. Initial aortic perfusion was with Marshall’s solution, followed by portal vein and hepatic artery perfusion with University of Wisconsin solution (UW). In DCDs, efforts were made to minimise warm ischemic time (defined as the period when the systolic blood pressure falls below 50 mmHg and the onset of cold perfusion) and cold ischemic time (from start of cold perfusion to reperfusion in the recipient). Liver transplantation was performed using a piggyback technique with initial reperfusion via the portal vein. Following transplantation all patients received immunosupressive therapy with Tacrolimus (Prograf) and prednisolone. Episodes of acute rejection were defined as an appreciable clinical manifestation (rise in AST) that was biopsy proven and responded to steroid therapy.

**APPAP staining procedures**

The second layer rabbit anti-mouse immunoglobulin (MR12/53) (DAKO Ltd) was incubated with heat-inactivated human AB serum to block anti-human Ig activity, and added to the sections for 30 minutes. After washing with Tris buffered saline, the third layer alkaline phosphatase-anti-alkaline phosphatase complex (AP7/6/7) (DAKO Ltd) was added for 30 minutes. The sections were washed with Tris buffered saline and enzyme activity was developed using Naphthol-As-MX (Sigma Aldrich Ltd, Poole, Dorset, UK) and Fast Red (Sigma Aldrich Ltd, UK) substrate for 20 minutes. The enzymatic reaction was stopped with a further wash with Tris buffered saline and counterstained with Gill’s hematoxylin (Sigma Aldrich Ltd, UK).

**Ceramide sample analysis**

Between 10 and 30 mg of tissue were obtained and transferred to a pre-weighed Eppendorf tube containing a steel bead. Then, 30 µL (per 10 mg of tissue) of a methanol: water mixture (4:1, containing the negative-mode internal standard heptadecanoic acid, 10µg/mL) was added and samples were homogenized for 5 min at 25 Hz (10 cycles of 0.5 min) in a TissueLyser (Qiagen, MD, US). Following this, 70 µl of the homogenate was added to an amber glass HPLC vial containing a 300 µl glass insert (Chromacol, UK). To this 200 µl of MTBE containing 10 µg/mL of positive-mode internal standard (tripentadecanoin) was added, and the samples were mixed via vortexing at room temperature for 60 min. Then, 30 µl of high purity water was added and samples were centrifuged at 2500 rpm for 20 min at 4 ºC. The upper lipid containing MTBE phase was then injected onto the LC-MS system directly from the vial. ). Quality control samples were created by pooling excess tissue homogenate (35 µl) from each biological sample; this pooling homogenate was then split into 70 µl aliquots for in-vial extraction.

In-vial dual extraction (IVDE) was performed for both positive and negative mode electrospray ionization-mass spectrometry (ESI-MS) analysis. LC-MS analysis of all samples was performed on a Waters ACQUITY Xevo-QToF UPLC-MS.

**Assessment of staining**

An increase in neutrophil infiltration of >2.5% was considered a significant increase after reperfusion. Increases in CD41 and P selectin expression on sinusoidal endothelium were often focal and increases of 2% of the total sinusoidal endothelium were considered a change following reperfusion.Expression of VCAM-1 was assessed as positive or negative on all structures within the liver and ICAM-1 and HLA class II were evaluated using a semi-quantitative scoring system. Expression of ICAM-1 on sinusoidal endothelium and hepatocytes was graded as follows: Grade 1, <70% of sinusoidal endothelium positive, hepatocytes negative; Grade 2, <90% of sinusoidal endothelium positive with occasional positive hepatocytes; Grade 3, > 90% of sinusoidal endothelium stained with multiple foci or extensive hepatocyte positivity. HLA class II antigen expression was assessed and graded as follows: Grade 1, endothelium of occasional sinusoids positive, hepatocytes negative; and Grade 2, multiple sinusoids with positive endothelium and occasional hepatocyte staining. Venous endothelium was always positively stained for vWF in all biopsies; however, there were variations in sinusoidal endothelium levels of vWF expression before and following reperfusion. Expression of vWF was evaluated using a semi-quantitative scoring system and was graded as follows: Grade 0, negative or occasional, isolated aggregations; Grade 1, weak sinusoidal staining; Grade 2, more extensive and intense staining of the sinusoidal endothelium. Blinded histologic analysis was performed by two independent observers. An increase in >1 grade after reperfusion was considered a significant increase in the level of expression.

| **Table A**. Identification of markers based on molecular weight, retention time and collision induced dissociation fragmentation of lipids. | | | | |
| --- | --- | --- | --- | --- |
| **m/z** | **Identified as** | **Observed ion** | **Fragments** | **RSD (%)**  **QC** |
| 582.51 | Cer (d18:1/16:0)/Cer16 | [M+FA-H]^-^ | 237 & 280 | 22.9 |
| 610.54 | Cer (d18:1/18:0)/Cer18 | [M+FA-H]^-^ | 237 & 308 | 14.3 |
| 638.57 | Cer (d18:1/20:0)/Cer20 | [M+FA-H]^-^ | 237 & 336 | 19.1 |
| 666.60 | Cer (d18:1/22:0)/Cer22 | [M+FA-H]^-^ | 237 & 364 | 4.93 |
| 694.64 | Cer (d18:1/24:0)/Cer24 | [M+FA-H]^-^ | 237 & 392 | 3.85 |

| **Table B**. Correlation of ceramides (n=23) to clinical outcomes (spearman correlations and Benjamini and Hochberg correction) ^**^q<0.01, ^***^q<0.001 | | | | | |
| --- | --- | --- | --- | --- | --- |
| **DCD-pre** | **C18-ceramide** | | **DCD-post** | **C18-ceramide** | |
|  | Corr. coefficient | q value |  | Corr. coefficient | q value |
| BIL 3 | 0.7747^**^ | 0.0031 | INR 2 | 0.7785^**^ | 0.0017 |
| BIL 9 | 0.8182^**^ | 0.0011 | INR 4 | 0.7565^**^ | 0.0028 |
| BIL 10 | 0.8252^**^ | 0.0010 | INR 8 | 0.8421^***^ | 0.0006 |
| BIL 11 | 0.9423^***^ | 0.00005 | CREAT 9 | 0.8476^***^ | 0.0005 |
| BIL 12 | 0.9000^***^ | 0.0009 | CREAT 10 | 0.8476^***^ | 0.0005 |
| BIL 13 | 0.9643^***^ | 0.0005 |  |  |  |
